# Comparison of Fracture Resistance of Endodontically Treated Premolar Restored With Composite Resin, Fiber Post, and Prefabricated Metal Posts With and Without Full Coverage Metal Crowns

**DOI:** 10.7759/cureus.117557

**Published:** 2026-10-06

**Authors:** Rahul Majumdar, Jitendra Lohar, Anoli J Hirani, Sangeeta Sangeeta, Hardeep Singh, Shantanu Singh

**Affiliations:** 1 Department of Conservative Dentistry and Endodontics, Pacific Dental College and Research Centre, Udaipur, IND; 2 Private Practitioner, Surendera Dental College and Research Institute, Sri Ganganagar, IND; 3 Private Practitioner, Dr. D. Y. Patil Dental College and Hospital, Pune, IND

**Keywords:** endodontically treated teeth (ett), fiber posts, fracture resistance, nayyar’s core, stainless steel metal posts

## Abstract

Background

Restoration of endodontically treated teeth (ETT) is challenging due to biomechanical changes and structural loss, with maxillary premolars being particularly susceptible to fracture. This study evaluated the influence of restorative design and loading direction on their fracture resistance.

Aim and objective

The objective of this study is to compare and evaluate the fracture resistance of endodontically treated premolar restored using composite resin, fiber post, and prefabricated metal posts with and without full-coverage metal crowns.

Methods

Sixty extracted human maxillary first premolars were prepared with MOD (mesial, occlusal, and distal) cavities and endodontically treated. Specimens were divided into three groups: Nayyar’s core, fiber posts, and stainless steel metal posts. Each group was subdivided into crowned and non-crowned restorations. Fracture resistance was measured under 90° (vertical) and 45° (oblique) loading using a universal testing machine. Intergroup comparisons were performed using one-way analysis of variance (ANOVA), while comparisons between 45° and 90° loading conditions were performed using paired t-tests. Data normality and homogeneity were assessed using the Shapiro-Wilk and Levene’s tests, respectively.

Results

Crown placement significantly improved fracture resistance across all restorative modalities (p<0.001). One-way ANOVA demonstrated significant differences among restorative subgroups at 45° and 90° loading (p<0.001). Paired comparisons demonstrated significantly higher fracture resistance under 90° loading than under 45° loading in all restorative subgroups (p<0.001).

Conclusion

Coronal coverage is the most critical factor for reinforcing ETT, providing superior resistance compared to non-crowned designs. While posts enhance strength in the absence of crowns, full-coverage restorations remain the most reliable method for protecting posterior ETT.

## Introduction

Careful biomechanical planning and functional performance are important when restoring endodontically treated teeth (ETT) [1]. At present, the current evidence indicates that the increased fracture risk of ETT is not caused by postroot canal changes in dentin properties, but rather by the considerable amount of tooth substance lost during access preparation, tooth preparation, and removal of caries during the root canal treatment [2].

The thin roots, steep cusp inclines and the exposure of the maxillary premolars to complex forces of mastication make them more susceptible to fracture. Marginal ridges removal, particularly in mesio-occluso-distal (MOD) cavity preparation, drastically compromises the rigidity of the tooth and creates the possibility of structural failure [3].

Adhesive composite restorations are effective in reinforcing a tooth with minimal structure loss, since they bond to enamel and dentin and enhance the distribution of stress and decrease cuspal flexure [4]. If the destruction of the coronal area is large, however, then additional support is usually needed. Posts are applied in these situations mainly to hold the core restoration in place and not for strengthening the tooth [5].

The mechanical characteristics of the post material are important for stress transmission. The modulus of elasticity of metal posts can be high and thus induce stress concentrations in the root [6]. For example, fiber-reinforced posts have mechanical properties more similar to dentin, which can lead to more even stress distribution and frequently to more desirable fracture patterns.

Full-coverage crowns are still the most effective of the options available when it comes to protecting the posterior ETT [7]. Cuspal coverage aids in the even distribution of occlusal forces, curtails crack propagation and improves resistance to functional loading. In general, oblique forces tend to increase fracture risk as they are more likely to create shear stresses and bending effects, while vertical forces tend to be better tolerated as they are parallel to the long axis of the tooth [8]. Thus, maintaining as much tooth structure as possible, choosing appropriate restorative material, and providing sufficient coronal protection for long-term clinical performance is essential to success in the restoration of ETT [9].

Therefore, the present study was carried out to compare and evaluate the fracture resistance of endodontically treated premolar restored with composite resin, fiber post and prefabricated metal post with and without full-coverage metal crowns.

## Materials and methods

Source of data

The maxillary first premolars (MP1) were collected from 60 orthodontic patients aged 18 to 24 years reporting to the Department of Oral and Maxillofacial Surgery, Pacific Dental College and Research Centre (PDCRC). After extraction, the teeth were washed and kept in 10% formalin at room temperature for further processing. The 60 specimens were randomly divided into three major experimental groups, with 20 specimens in each group.

Ethical approval

The study protocol was approved by the Institutional Ethics Committee, Pacific Dental College and Research Centre, before the research was started. All the procedures were performed in line with accepted in vitro dental research ethical guidelines.

Inclusion and exclusion criteria

The study included sound, intact human maxillary first premolars with fully developed roots and complete apical formation that had been extracted for orthodontic purposes. Teeth were excluded if they exhibited fracture lines or cracks, radiographic evidence of calcification, internal or external root resorption, developmental anomalies, caries, or existing restorations.

Armamentarium

The materials and equipment used in the study included a dental operating microscope (Labomed Prima, Labo America Inc., Fremont, CA), ProTaper Gold rotary file system (Dentsply Sirona India Pvt. Ltd, Noida, India), 3% sodium hypochlorite solution, gutta-percha cones, AH Plus root canal sealer (Dentsply), Beautibond bonding agent (Shofu Dental India, New Delhi, India), nanohybrid composite resin (Tetric N-Ceram, Ivoclar Vivadent, Gurugram, India), Tenax Fiber White posts TF #11 (Coltene), ParaPost stainless steel posts #3 (Coltene, Altstätten, Switzerland), Tenax drill TE-A-11, ParaPost drill size 4.5, cold-cure acrylic resin (DPI-RR, Dental Products of India, Mumbai, India), polysiloxane impression material (Dentsply), Calamus obturation unit (Dentsply Maillefer, Ballaigues, Switzerland), and a universal testing machine.

Methodology

Sixty extracted maxillary first premolars were initially examined under 15× magnification using a dental operating microscope to confirm the absence of caries, restorations, calcifications, root resorption, cracks, and other structural defects. Standardized MOD cavity preparations and endodontic access cavities were prepared in all specimens. Root canal instrumentation was performed using the ProTaper Gold rotary system up to file size F2 with copious irrigation using 3% sodium hypochlorite solution (Figure 1).

**Figure 1:**
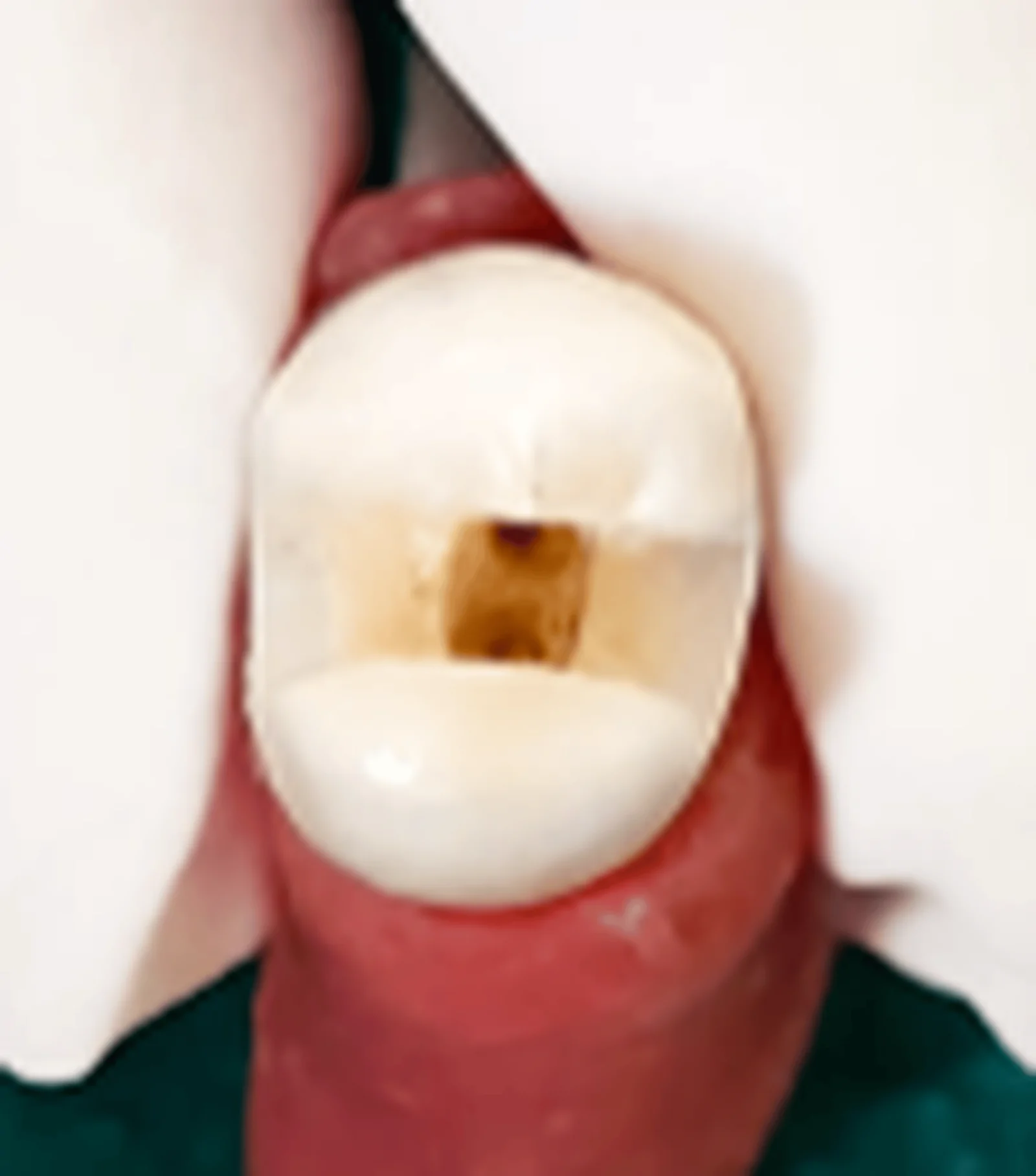
Standardized mesio-occluso-distal (MOD) access cavity preparation in an extracted maxillary first premolar before post-endodontic restoration.

Following biomechanical preparation, all canals were obturated using gutta-percha and AH Plus sealer by the continuous wave compaction technique. The specimens were then divided into three groups according to the post-endodontic restoration employed.

Group 1 consisted of specimens restored using Nayyar’s core technique with composite resin. This group was further subdivided into Group N (without crown) and Group NC (with crown). Approximately 3 mm of coronal gutta-percha was removed using a heated spreader, followed by restoration with Beautibond adhesive and nanohybrid composite resin.

Group 2 consisted of specimens restored with fiber posts and composite resin cores, subdivided into fiber post with composite core without crown and fiber post with composite core with crown.

**Figure 2:**
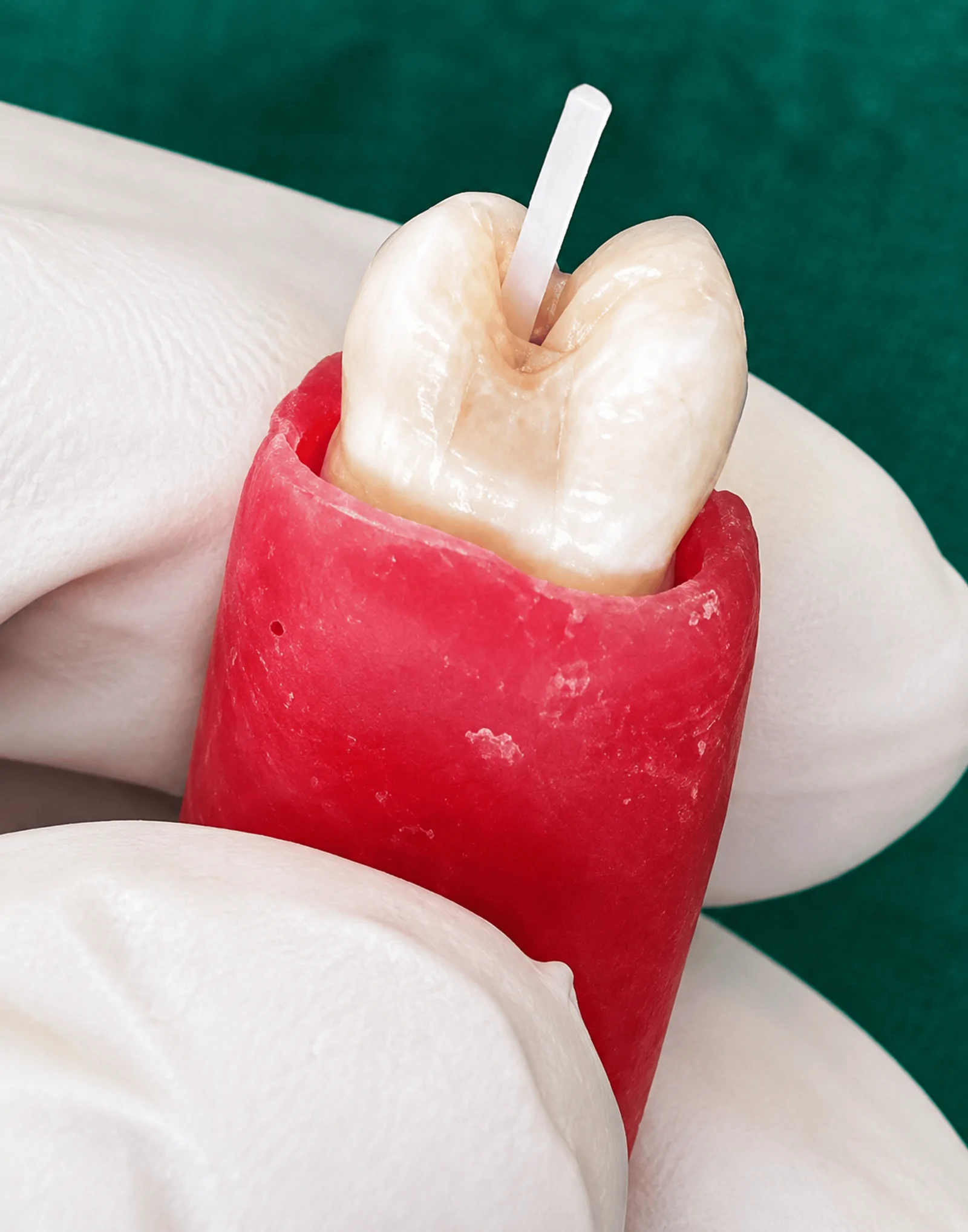
Placement of the fiber post within the prepared palatal canal of an endodontically treated maxillary first premolar.

Group 3 consisted of specimens restored with prefabricated stainless steel metal posts and composite resin cores, subdivided into metal post with composite core without crown and metal post with composite core with metal crown (Figure 3).

**Figure 3:**
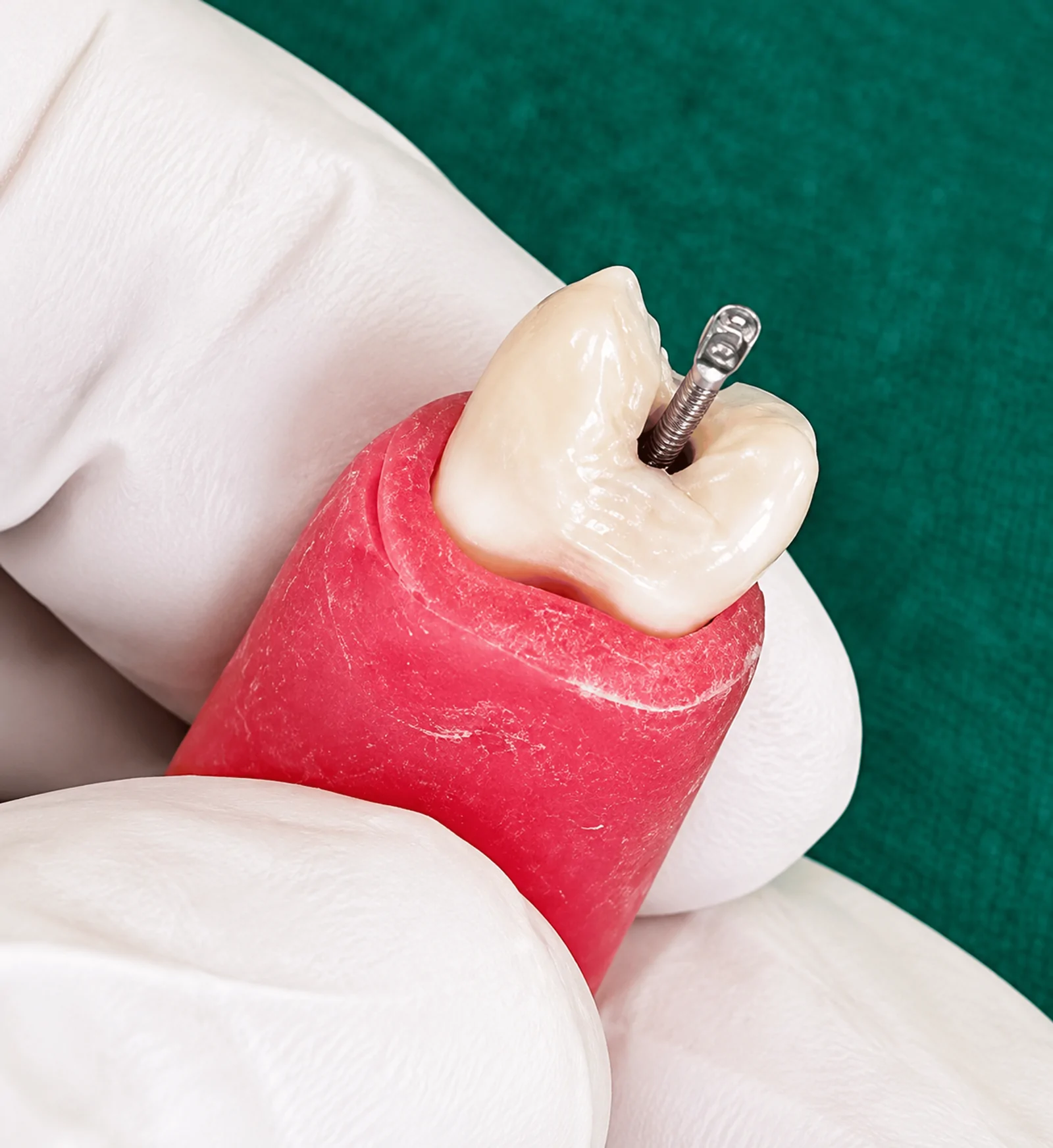
Placement of a prefabricated stainless steel metal post within the prepared palatal canal of an endodontically treated maxillary first premolar.

For the fiber post and metal post groups, post-space preparation was performed using the respective manufacturer-recommended drills, ensuring the preservation of a 5 mm apical gutta-percha seal. Fiber post spaces were prepared using the Tenax drill TE-A-11, whereas metal post spaces were prepared using ParaPost drill size 4.5.

Each specimen was embedded in cold-cure acrylic resin blocks measuring 25 mm×20 mm×20 mm, with the root positioned up to 2 mm below the cementoenamel junction. To simulate the periodontal ligament, the root surfaces were coated with a thin layer of polysiloxane impression material before embedding. The specimens were allowed to cure undisturbed for 24 hours.

The mounted teeth were subjected to fracture resistance testing using a universal testing machine (Figure 4). Loads were applied at 90° and 45° angles using a 3 mm diameter spherical indenter at a crosshead speed of 0.5 mm/min until fracture occurred (Figure 5).

**Figure 4:**
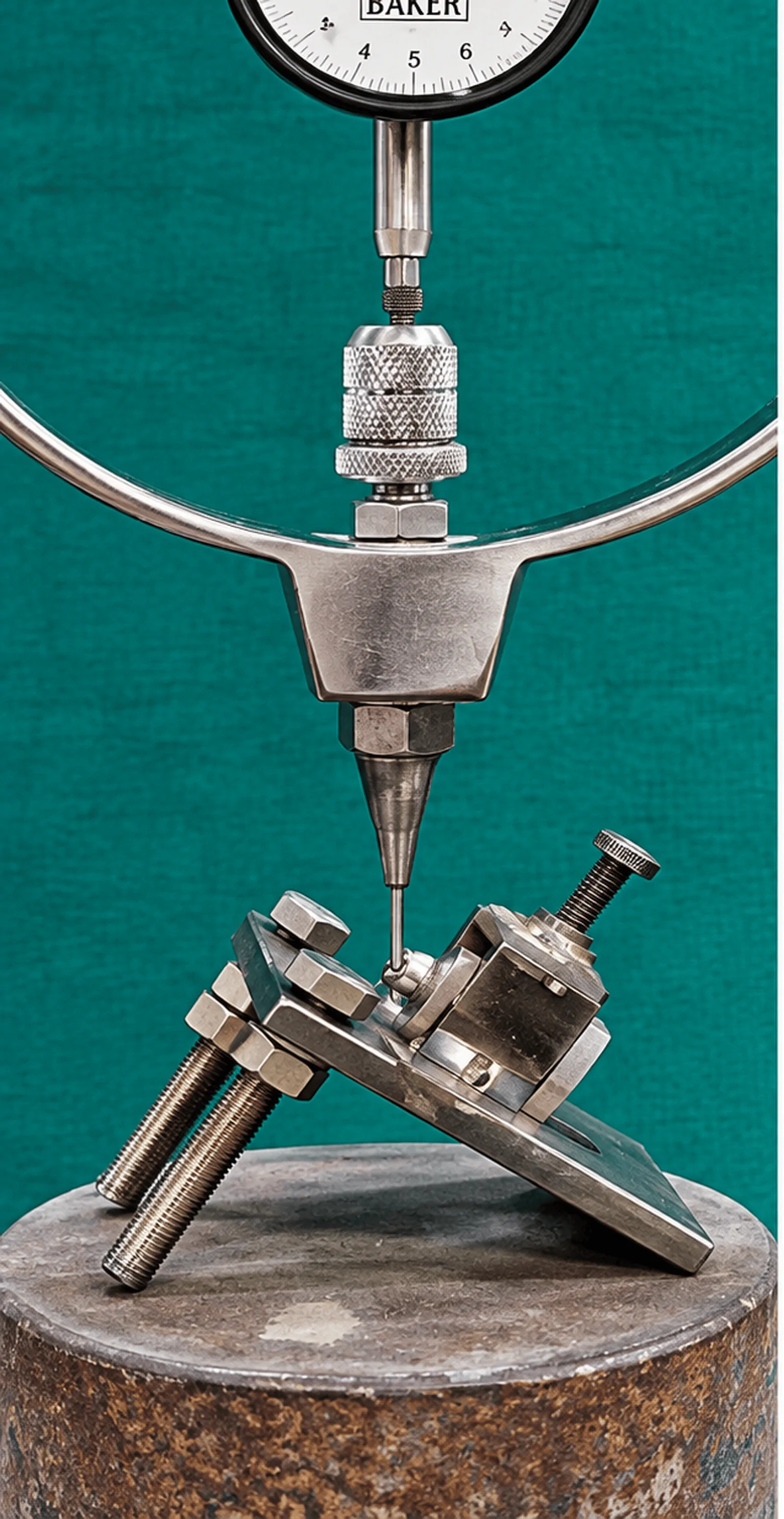
Endodontically treated maxillary first premolar specimen subjected to fracture resistance testing under a 45° oblique loading configuration using a universal testing machine.

**Figure 5:**
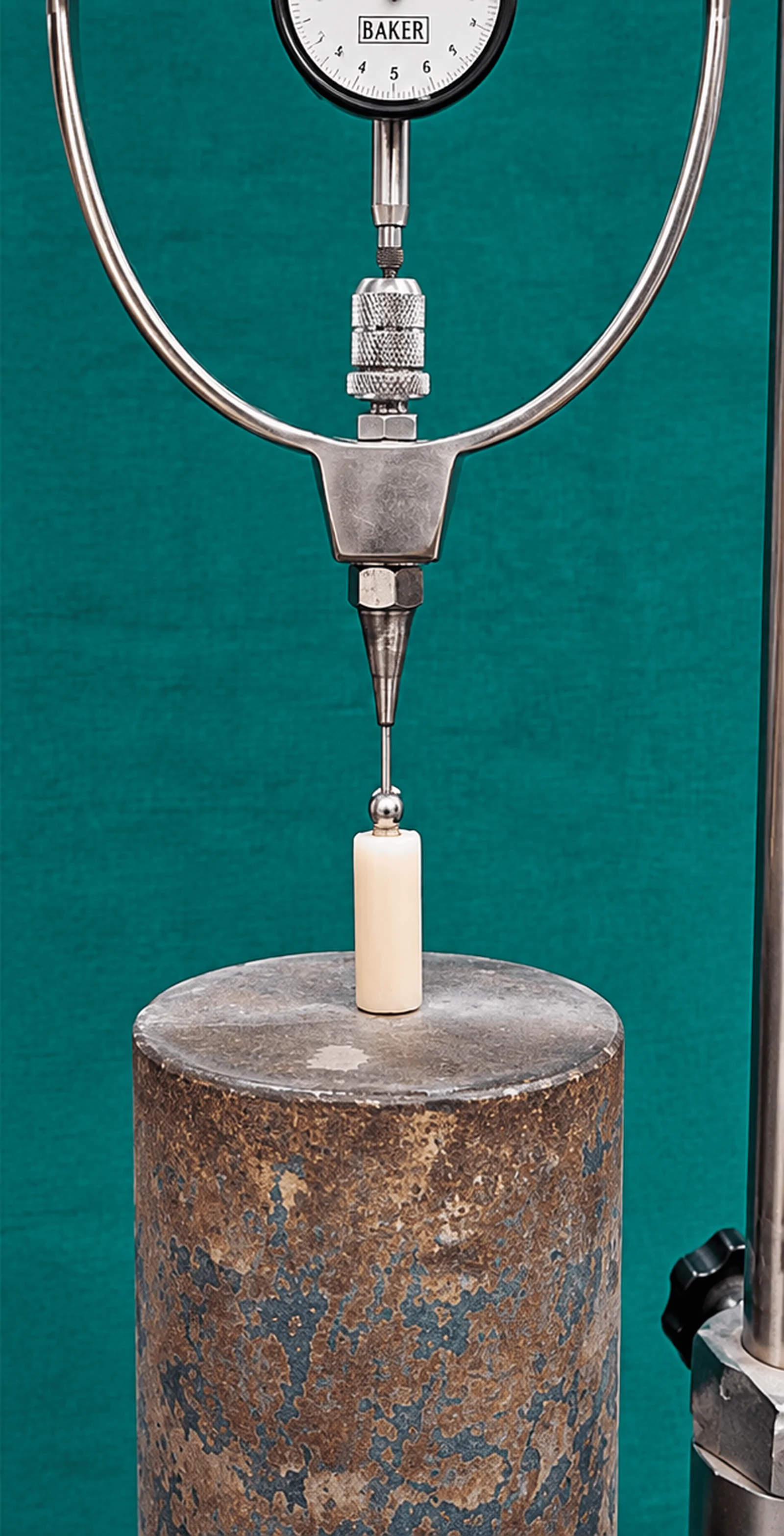
Endodontically treated maxillary first premolar specimen subjected to fracture resistance testing under a 90° vertical loading configuration using a universal testing machine.

Data were entered into Microsoft Excel 2010 (Microsoft, Redmond, WA) and analyzed using SPSS version 21.0 (IBM Corp, Armonk, NY). Descriptive statistics were expressed as mean and standard deviation. Intergroup comparisons were performed using one-way analysis of variance (ANOVA), followed by appropriate post-hoc comparisons where applicable. Comparisons between 45° and 90° loading conditions were performed using paired t-tests.

## Results

Under 45° oblique loading, restorations without crowns demonstrated considerably lower fracture resistance than crowned restorations (Table 1). Among the non-crowned groups, metal posts showed the highest mean fracture resistance (1258.6±280.2 N), followed by fiber posts (1125.4±210.7 N), whereas Nayyar’s core exhibited the lowest value (640.2±120.5 N). In contrast, crown placement markedly increased fracture resistance in all restorative groups. The highest mean value was observed in the Nayyar’s core with crown subgroup (2105.6±240.3 N), followed by the metal post with crown (1955.3±390.5 N) and fiber post with crown (1850.9±260.8 N). One-way ANOVA demonstrated a statistically significant difference among the restorative subgroups (F=47.18, p<0.001).

**Table 1:** Descriptive statistics of fracture resistance (N) at 45° loading. Differences among restorative subgroups were assessed using one-way analysis of variance (ANOVA).

Group	Subtype	Minimum (N)	Maximum (N)	Mean (N)	SD (N)	F-value	p-value
Group 1	Nayyar’s Core without Crown	480	980	640.2	120.5	47.18	<0.001
Group 1	Nayyar’s Core with Crown	1750	2550	2105.6	240.3		
Group 2	Fiber Post without Crown	820	1450	1125.4	210.7		
Group 2	Fiber Post with Crown	1500	2250	1850.9	260.8		
Group 3	Metal Post without Crown	900	1600	1258.6	280.2		
Group 3	Metal Post with Crown	1600	2400	1955.3	390.5		

Intragroup analysis revealed a highly significant increase in fracture resistance following crown placement across all restorative modalities. The greatest improvement was observed in the Nayyar’s core group, which showed an increase of 1465.4 N after crown placement (p<0.001). Fiber and metal post groups also demonstrated significant improvements, with mean increases of 725.5 N and 696.7 N, respectively (p<0.001).

Intergroup comparison under 45° loading without crowns demonstrated a statistically significant difference among the three restorative systems (F=23.12, p<0.001) (Table 2). Fiber and metal posts demonstrated higher mean fracture resistance than Nayyar’s core, whereas the difference between fiber and metal posts was comparatively smaller (Table 2).

**Table 2:** Intergroup Comparison Of Fracture Resistance (N) At 45 Degree Loading (Without Crown) F=23.12, p<0.001 Statistical test: One-way analysis of variance (ANOVA).

Group	Subtype	Mean (N)	SD (N)	SE (N)	Minimum (N)	Maximum (N)	F-value	p-value
Group 1	Nayyar’s Core	640.2	120.5	38.1	480	980	23.12	<0.001
Group 2	Fiber Post	1125.4	210.7	66.6	820	1450		
Group 3	Metal Post	1258.6	280.2	88.6	900	1600		

Under 90° vertical loading, all groups demonstrated higher fracture resistance values compared with oblique loading (Table 3). Among non-crowned restorations, metal posts exhibited the highest mean fracture resistance (1525.4±260.8 N), followed by fiber posts (1450.7±230.6 N) and Nayyar’s core (980.5±150.2 N). Crowned groups again showed substantially greater resistance, with Nayyar’s core plus crown demonstrating the highest mean value (2555.8±310.4 N), followed by metal post with crown (2320.6±360.7 N) and fiber post with crown (2255.3±290.1 N). One-way ANOVA demonstrated a statistically significant difference among the restorative subgroups (F=50.30, p<0.001).

**Table 3:** Descriptive statistics of fracture resistance (N) at 90° loading. Differences among restorative subgroups were assessed using one-way analysis of variance (ANOVA). F=50.30, p<0.001

Group	Subtype	Minimum (N)	Maximum (N)	Mean (N)	SD (N)	F-value	p-value
Group 1	Nayyar’s Core without Crown	700	1300	980.5	150.2	50.30	<0.001
Group 1	Nayyar’s Core with Crown	2200	3000	2555.8	310.4		
Group 2	Fiber Post without Crown	1100	1800	1450.7	230.6		
Group 2	Fiber Post with Crown	2000	2700	2255.3	290.1		
Group 3	Metal Post without Crown	1200	1900	1525.4	260.8		
Group 3	Metal Post with Crown	2100	2800	2320.6	360.7		

Intergroup analysis under vertical loading mirrored the findings observed under oblique loading. Fiber and metal posts significantly outperformed Nayyar’s core in the absence of crowns, whereas no significant difference was found between fiber and metal post systems. In crowned specimens, fracture resistance values were largely comparable, although Nayyar’s core with crown demonstrated slightly superior performance (Table 4).

**Table 4:** Comparison of fracture resistance between 45° and 90° loading conditions among the restorative subgroups. Paired t-tests were used to compare fracture resistance between the two loading conditions.

Group	Subtype	45° Mean±SD (N)	90° Mean±SD (N)	Mean Difference (N)	t-value	p-value	Significance
Group 1	Nayyar’s Core without Crown	640.2±120.5	980.5±150.2	+340.3	8.74	<0.001	Highly significant
Group 1	Nayyar’s Core with Crown	2105.6±240.3	2555.8±310.4	+450.2	7.92	<0.001	Highly significant
Group 2	Fiber Post without Crown	1125.4±210.7	1450.7±230.6	+325.3	9.16	<0.001	Highly significant
Group 2	Fiber Post with Crown	1850.9±260.8	2255.3±290.1	+404.4	8.21	<0.001	Highly significant
Group 3	Metal Post without Crown	1258.6±280.2	1525.4±260.8	+266.8	6.87	<0.001	Highly significant
Group 3	Metal Post with Crown	1955.3±390.5	2320.6±360.7	+365.3	6.94	<0.001	Highly significant

A direct comparison between loading directions revealed significantly higher fracture resistance under 90° loading than under 45° loading for all restorative groups (p<0.001). The increase ranged from 266.8 N to 450.2 N, indicating that vertical forces were better tolerated than oblique forces irrespective of restorative design.

## Discussion

In the present study, the fracture resistance of endodontically treated teeth (ETT) restored with Nayyar's core, fiber posts and metal posts were compared and evaluated in oblique (45°) and vertical (90°) loading conditions with and without full-coverage crowns. The results show that the fracture resistance is dependent on restorative design, post‐core configuration, crown coverage, and loading direction, highlighting the need for a combination of these factors to affect the mechanical properties of ETT.

One of the key findings was that the fracture resistance of full-coverage crowns was significantly improved after the placement of a full-coverage crown, across all restorative groups. Cuspal coverage resulted in a greater resistance to fracture in crowned teeth than non-crowned teeth, suggesting the protective effect of cuspal coverage in structurally deficient teeth. This observation is similar to Aquilino and Caplan (2002), who found a significant correlation between crown placement and long-term survival of endodontically treated teeth; teeth that were not crowned after obturation had a significantly higher rate of tooth loss as compared to those that were crowned after obturation [10]. Likewise, Sorensen and Martinoff (1984) stressed the role of coronal coverage and the amount of remaining tooth structure in assessing the prognosis for endodontically treated teeth [11]. In addition, Scotti et al. (2011) showed that the coverage of the cusp is a key factor in decreasing the fracture risk of endodontically treated maxillary premolars [12]. All the results presented here support the theory that preservation and protection of the remaining coronal tooth structure may be a more critical factor than the post system itself.

The within-group analysis also showed a significant increase in fracture resistance after crown placement. The biggest improvement was seen in the Nayyar's core group where a conservative coronal-radicular core could work well if it is properly supported by a full-coverage restoration. This finding is in accordance with the study conducted by Abduljabbar et al. (2012) to assess fracture resistance of various post-core system under all-ceramic crowns, and the study showed that the interaction between the post-core system and the definitive crown restoration has an effect on fracture resistance [13]. Similarly, Jiangkongkho et al. (2013) reported improved fracture resistance with the use of a ferrule and full-coverage crown versus not adding the protective elements [14]. The findings of the present study therefore highlight the need to understand the role of coronal reinforcement and circumferential protection instead of the post as an individual reinforcement.

The metal-post and fiber-post groups of specimens had higher fracture resistance than the Nayyar's core group, but there was no statistically significant difference between these two groups. The results of the present study suggest that, with the experimental conditions used in the present study, fracture resistance may not be the primary factor in determining the post. Other factors such as the amount of remaining tooth structure, core material, post geometry, cementation, and adhesive interface could significantly affect the obtained fracture load. Alshahrani et al. (2020) showed how fracture resistance of endodontically treated teeth restored with fiber posts is affected by the properties of the core and luting material [15]. More recently, Aly et al. (2025) also pointed out that fracture resistance is multifactorial and is a function of the remaining tooth structure, post material, post diameter and post design [16]. The present study does not detect a significant difference between the fiber and metal post groups, and these observations make a plausible explanation for this.

Chhabra et al. (2022) also showed a good fracture resistance in customized glass-fiber post restorations in endodontically treated maxillary premolars [17]. Biswas et al. (2025) also identified variations in fracture resistance of different esthetic post systems, highlighting the significance of post design and characteristics of the underlying tooth substance [18]. Hence, it may not always be assumed that equivalent fracture loads are equal to comparable biomechanical performance, as fracture mode and reparability should always be taken into account.

In all the restorative groups, specimens subjected to vertical loading demonstrated higher fracture resistance than those subjected to oblique loading. Biomechanically, this difference is attributed to oblique forces, which induce a combination of compressive, tensile and shear stresses, and can cause stress concentrations in the cervical region and at the root/restoration interface, leading to initiation and propagation of cracks. In contrast, vertical loading is mainly compressive and can lead to a more favorable stress distribution along the long axis of the tooth. Eskitaşcioğlu et al. (2002) tested post-core systems with a 45° fracture-strength test and finite-element stress analysis and found that the direction and distribution of applied forces are significant factors that influence the stress pattern of the restored teeth [19]. 

In the present study, an important finding was that the combination of Nayyar's core with a full coverage crown performed well. This is in agreement with the original concept of Nayyar et al. (1980), who suggested an amalgam coronal-radicular dowel and core for restoring endodontically treated posterior teeth [20]. Based on the results of the present study, the conservative post-core treatment could provide a significant amount of fracture resistance without extensive preparation of the radicular area, when enough coronal protection is available. However, the choice of a restorative design must be dependent upon the amount and configuration of remaining tooth structure, the presence of a ferrule effect, preparation geometry, the type of restorative material, and the type of adhesive strategy. The authors of another study, Kazemi-Yazdi et al. (2023), also showed that the preparation design has a significant effect on the fracture resistance of maxillary premolars restored with endocrowns [21]. Therefore, the present results confirm the tooth-structure-oriented approach in which preservation of sound dentin and sufficient protection of the coronal dentin is prioritized instead of using more rigid post systems routinely.

## Conclusions

The restoration of ETT is still a real challenge because of the structural and biomechanical changes that take place after endodontic treatment, such as the loss of tooth structure, dentin dehydration and change in stress distribution. Based on the limitations of the present in vitro study, the following factors were found to be the most significant factors affecting fracture resistance of endodontically treated teeth: coronal coverage, restorative design, post type, and direction of loading. The most reinforcement was found with full coverage crowns. Fiber posts exhibited fracture resistance comparable to metal posts.
